# Effect of Gamma-Ray Irradiation on the Growth of Au Nano-Particles Embedded in the Germano-Silicate Glass Cladding of the Silica Glass Fiber and its Surface Plasmon Resonance Response

**DOI:** 10.3390/s19071666

**Published:** 2019-04-08

**Authors:** Seongmin Ju, Won-Taek Han

**Affiliations:** School of Electrical Engineering and Computer Science, Gwangju Institute of Science and Technology, Gwangju 61005, Korea; jusm@gist.ac.kr

**Keywords:** γ-ray irradiation, surface plasmon resonance, fiber sensor, nano-particles, cladding embedded optical fiber

## Abstract

The effect of γ-ray irradiation on the surface plasmon resonance (SPR) sensing capability of refractive index (n = 1.418–1.448) of the silica glass optical fiber comprised of germano-silicate glass cladding embedded with Au nano-particles (NPs) was investigated. As the γ-ray irradiation increased from 1 h to 3 h with the dose rate of 1190 Gy/h, the morphology of the Au NPs and the SPR spectrum were found to change. The average diameter of Au NPs increased with the aspect ratio from 1 to 2, and the nano-particles became grown to the clusters. The SPR band wavelength shifted towards a longer wavelength with the increase of total dose of γ-ray irradiation regardless of the corresponding refractive indices. The SPR sensitivities (wavelength/refractive index unit, nm/RIU) also increased from 407 nm/RIU to 3553 nm/RIU, 1483 nm/RIU, and 2335 nm/RIU after the γ-ray irradiation at a total dose of 1190 Gy, 2380 Gy, and 3570 Gy, respectively.

## 1. Introduction

Transparent host materials embedded with noble metal nano-particles (NPs) such as Au and Ag are of great interest due to their unique characteristics of surface plasmon resonance (SPR) arising from the excitation of electron density oscillations around metal NPs [1,2,3,4,5,6,7,8,9]. The localized SPR usually observed by confined colloidal, periodic, nano-systems gets resonantly excited when the wavelength of incident light is equal to the characteristic wavelength of metal NPs [10,11,12,13]. This comes from confined conduction electrons oscillating in resonance with the electromagnetic field. The localized SPR of metal nanostructures seems much more suitable for spectral tunability and strong enhancement of the local electric field [14]. Thus, the localized SPR has been widely used in the sensing of chemical, physical, and biological quantities based on the change of the refractive index [15,16,17,18,19,20,21,22,23].

The position and intensity of the SPR band depend on the size, shape, and inter-particle separation of the NPs and the dielectric property of the matrix surrounding the NPs [3,4,7,24,25,26,27,28,29,30,31,32,33]. The position of SPR peak is known to shift towards long or short wavelengths upon the change in NPs size. Therefore, various fabrication methods of metal NPs with different geometries such as nano-spheres, nano-rod, nano-shells, nano-tubes, nano-cubes, nano-wires, etc. have been proposed to increase the sensitivity and usability of the SPR sensors [7,24,26,27,28,29,30,32,33,34,35,36,37,38]. Among these NPs, for the strong absorption of incident light followed by field enhancement of the surface of Au NPs, spherical Au NPs are more favorable because of their isotropic structures which allow coupling to occur in every direction, rather than only in one direction of Au NPs [39,40]. However, even though spherical NPs have strong SPR absorption, there is a limit in terms of amplitude increase and spectral tunability in the visible–NIR region. Also, a single strong SPR spectral feature usually deviates when the eccentricity of the particles increases [41]. Because of these reasons, longitudinal SPR absorption by controlling the aspect ratio of Au NPs has attracted much attention for its large sensitivity and spectral tunability [42]. 

Various post-processing techniques of the irradiation by the pulsed laser, the charged ion, and the γ-ray have been investigated to control NPs with desired size and shape [13,43,44,45,46,47,48,49,50]. Our group has reported a heat treatment method to control the size of NPs in glass fiber [51,52]. Among such post-irradiation processing techniques, the high energy irradiation was proven to control effectively the size, shape, and spacing of the NPs by creating ion tracks resulting from Coulomb explosion and/or thermal spikes accompanying the excitations [53,54,55,56]. Due to the passage of a swift heavy ion, the particles grow and combine into clusters leading to chemical, physical, and optical changes [57].

Recent advances in the SPR application have led to the realization of the optical fibers incorporated with metal NPs and the fibers coated with metal thin layers [58,59,60,61,62,63,64,65,66]. In the SPR sensor based on the optical fiber, an evanescent wave is formed by the interface between the surface of the optical fiber and the metal NPs embedded or coated on the surface. The fiber-optic SPR sensor is an alternative to overcome the disadvantages of previous conventional prism-based SPR sensors because of its attractive advantages of simple and flexible design, compactness, and remote sensing capability for all-optical applications. Especially, the development of a fiber-optic localized SPR sensor based on noble metal NPs has attracted increasing attention, because it provides several advantages over the use of continuous metal thin films, such as the ease of fabrication, modification and control and the high sensitivity due to the increased surface area of metal/glass interface [67,68]. The fiber-optic localized SPR sensor for label-free biochemical sensors simply measures the change in absorption spectra by sweeping broadband light through NPs in the fiber [68,69]. Recently, we have reported the fiber-optic refractive index sensor using the novel optical fiber incorporated with Au NPs in the cladding [52,70]. In this paper, we report new results of the effect of γ-ray irradiation on the SPR sensing capability by inducing the morphological change in Au NPs embedded in the fiber.

## 2. Experiments

### 2.1. Optical Fiber for Surface Plasmon Resonance Sensor

The fabrication process of the germano-silicate optical fiber embedded with Au NPs in the cladding and the measurement of its SPR sensing property was described in detail in our previous work [52,70,71]. The Au NPs(cladding)-doped glass fiber coated with low refractive index polymer (SSCP Co., Ltd., Ansan-si, Kyunggi-do, South Korea, FIRON UVF PC-375, n = 1.3820 @ 852 nm) was designed to enable a light to propagate into the cladding, not into the core. Note that the refractive index of the cladding was larger than that of the core and the polymer coating. The refractive index difference between the cladding and the core (∆n_cladding–core_) and that between the cladding and the coating ((∆n_cladding–coating_) were 0.0015 and 0.0764, respectively. The cladding width and total diameter of the optical fiber were 2.6 μm and 124.3 μm, respectively. Thus, surface plasmon waves are induced around Au NPs in the cladding of the fiber by a light wave traveling through the cladding.

The cladding width was designed and optimized by considering the launching efficiency of light and the subsequent SPR sensing efficiency. If the cladding containing Au NPs is large over the optimized width under the assumption that the concentration of Au NPs as well as size, shape, and inter-particle separation of the NPs is constant, the launching efficiency of light into the cladding of the fiber definitely increases, but the SPR sensing efficiency will not be affected much due to the increase of the distance from the fiber surface to the sensing objects. On the other hand, if the cladding is thin, the concentration of Au NPs per unit area increases, but less light goes through the cladding of the fiber and thus the SPR sensing efficiency will not be affected either.

### 2.2. Verification of Existence and Morphology of Au Nano-Particles

To investigate the effect of γ-ray irradiation on change in morphology of Au NPs and the SPR sensing capability, the fabricated fiber was irradiated by γ-ray from a ^60^Co radiation source (Nordion Inc., Ottawa, ON, Canada, MSD Nordion, pencil type/C-188 sealed) with the dose rate of 1190 Gy/h for 1 h to 3 h at room temperature in air. The γ-ray doses were measured using the alanine pellet dosimeter and estimated by electron paramagnetic resonance analysis with BRUKER’s e-scan alanine dosimetry system (Bruker Inc., Billerica, MA, USA). Then to confirm the formation and the change in size and distribution of Au NPs, the samples of the fibers with and without the γ-ray irradiation were examined by transmission electron microscopy (TEM; FEI Co., Hillsboro, OR, USA, Technai^TM^ G^2^ F30 S-Twin 300 KeV), which were prepared after flaking the surface of the fiber in the longitudinal direction by the dual beam focused ion beam (FIB; FEI Co., Hillsboro, OR, USA, Helios NanoLab^TM^ FIB 600). Optical absorption of the fibers was measured to verify again the existence of Au NPs by the cut-back method using the optical spectrum analyzer (OSA; Ando Electric Co., Ltd., Kanagawa Kawasaki-shi, Kanagawa-ken, Japan, AQ 6315B) and white light source (WLS; Ando Electric Co., Ltd., Kawasaki-shi, Kanagawa-ken, Japan AQ 4305). Note that the variation of the input light signal is eliminated because only the change in the length of the fiber is measured by the cut-back method. 

### 2.3. Surface Palsmon Resonance Measurement

**Figure 1:**
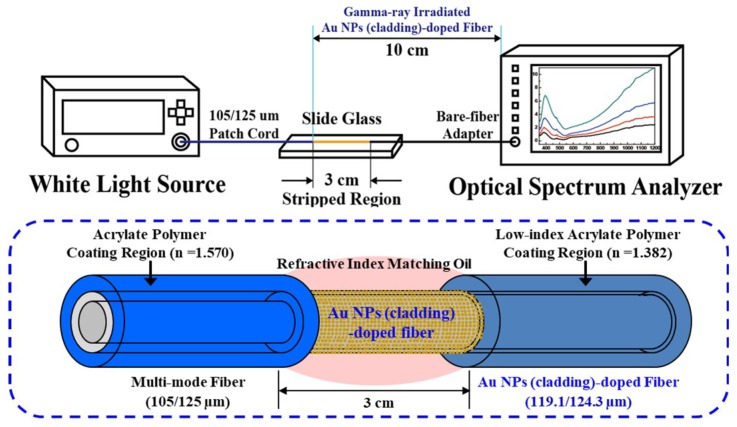
Schematic diagram of the SPR measurement set-up using the optical fiber incorporated with Au NPs in the cladding.

To characterize SPR sensing property, the change in optical absorption by the γ-ray irradiation was measured by putting small drops of the refractive index matching oil with various refractive indices (n = 1.418–1.448, Cargille Labs, Cedar Grove, NJ, USA) on the surface of the stripped portion (3 cm) of the 10 cm fiber (Figure 1). The coated polymer of the irradiated fiber was stripped off using acetone and the fiber was spliced with a commercial multi-mode fiber (diameters of core, cladding, coating were 105, 125, and 250 μm, respectively). The light was launched into the commercial multi-mode fiber with FC/PC-type connector for stable light supply and the resonance wavelength was measured by directly coupled to the OSA using a bare-fiber adapter without splicing with another fiber in order to minimize the signal distortion or intensity degradation at the other end of the fiber. Note that the change in optical absorption of the fiber without γ-ray irradiation was measured by using the fiber of 20 cm total length with the surface of the stripped portion (3 cm) according to the optimized fiber length for SPR sensor implementation derived from previous experimental results [70]. 

## 3. Results

**Figure 2:**
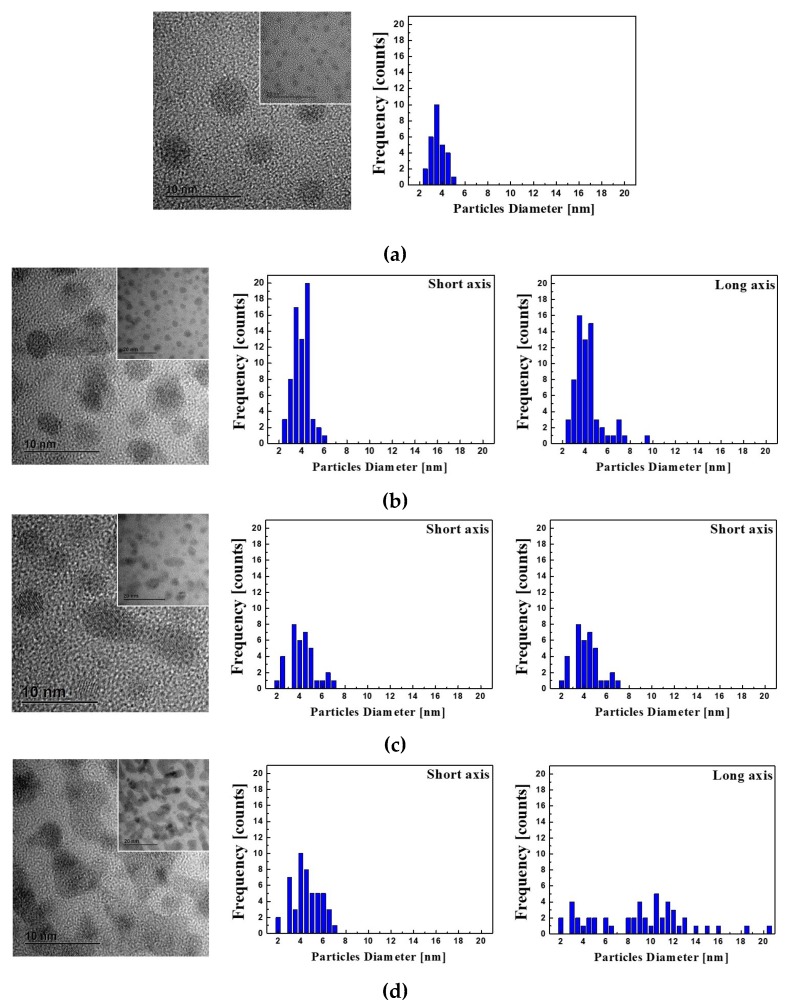
TEM images and size distribution of the major and minor axis lengths of the Au NPs incorporated in the cladding of the fiber (**a**) before and after the γ-ray irradiation at the total dose of (**b**) 1190 Gy, (**c**) 2380 Gy, and (**d**) 3570 Gy.

TEM images and size distributions of Au NPs in the cladding of the fiber before and after the γ-ray irradiation are shown in Figure 2. The morphology of the Au NPs was dramatically changed with the increase of the γ-ray irradiation dose level from 0 Gy to 1190 Gy, 2380 Gy, and 3570 Gy. Before the γ-ray irradiation, the crystalline Au NPs were spherical and uniformly distributed with average diameter of 3.8 nm (2.5 nm~5.2 nm) with the aspect ratio of 1.00. After irradiation, most of the Au NPs seemed to aggregate and became clusters. Under the total dose of γ-ray irradiation with 1190 Gy, 2380 Gy, and 3570 Gy, the short axis lengths was 4.0 nm (size distribution: 2.3 nm~5.8 nm), 4.2 nm (size distribution: 2.0 nm~6.8 nm), and 4.5 nm (size distribution: 1.8 nm~6.9 nm), respectively, and the long axes lengths were 4.2 nm (size distribution: 2.3 nm~9.6 nm), 5.4 nm (size distribution: 2.0 nm~12.7 nm), and 9.0 nm (size distribution: 1.8 nm~20.7 nm), respectively. 

**Figure 3:**
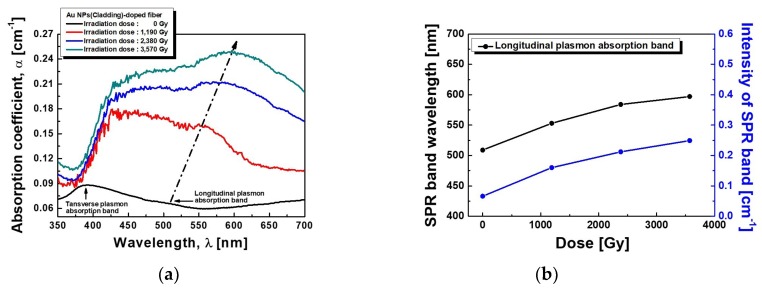
(**a**) Absorption spectra and (**b**) peak wavelength and intensity of the SPR band of the optical fiber incorporated with Au NPs before and after the γ-ray irradiation at the total dose of 1190 Gy, 2380 Gy, and 3570 Gy.

To verify the existence of Au NPs in the fiber cladding again, optical absorption was measured by the cut-back method. Figure 3a compares the optical absorption spectra of the fibers before and after the γ-ray irradiation at the total dose of 1190 Gy, 2380 Gy, and 3570 Gy. Before irradiation, the absorption bands due to SPR were found to appear peaking at 392 nm (absorption coefficient α = 0.088 cm^−1^) and 509 nm (α = 0.066 cm^−1^), which depended on the particle size of Au NPs in the cladding of the fiber [5,8,52,72]. After the γ-ray irradiation, the intensity and the peak wavelength of the two SPR bands were found to change. While the second SPR band at a longer wavelength of 509 nm has clearly shown the increase of its intensity and wavelength shift, the first SPR band at 392 nm has not shown any significant increase of its intensity and wavelength shift. With the increase of γ-ray irradiation dose, the splitting of the SPR bands became more distinct [2,59,60,61]. The intensity of the second absorption band at 509 nm corresponding to the interparticle plasmon coupling of the aggregated Au NPs at the total dose of 1190 Gy increased from α = 0.160 cm^−1^ to α = 0.212 cm^−1^, and α = 0.249 cm^−1^ at the total dose of 2380 Gy and 3570 Gy, respectively. Note that the absorption band around 392 nm, which cannot be distinguished whether it is corresponding to spherical Au NPs or the radiation-induced defects by the γ-ray irradiation, at the total dose of 1190 Gy also increased from α = 0.178 cm^−1^ to α = 0.204 cm^−1^ and α = 0.226 cm^−1^ with total doses of 2380 Gy and 3570 Gy, respectively.

**Figure 4:**
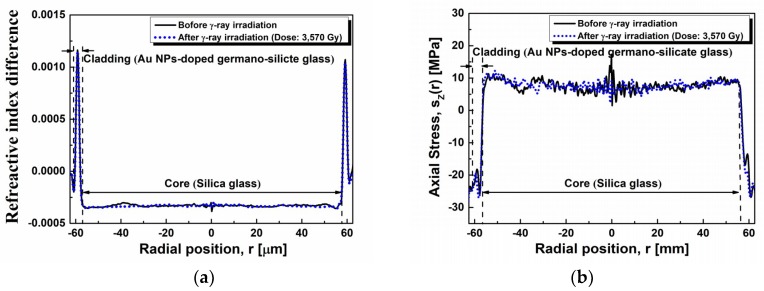
(**a**) Refractive index difference and (**b**) stress profile of the fiber before and after the γ-ray irradiation with the dose rate of 3570 Gy.

Considering that the change of the refractive index and the residual stress of the fiber induced by the γ-ray irradiation may affect the transmission characteristics of the optical signal, the refractive index and the residual stress of the fiber before and after the γ-ray irradiation of the total dose of 3570 Gy were measured by the fiber index profiler (Interfiber Analysis, Sharon, MA, USA, IFA-100) as shown in Figure 4. Before the γ-ray irradiation, the refractive index (∆n_cladding–core_ = 0.0015, with the standard deviation of 1.05 × 10^−4^) of the cladding larger than that of the core was due to the presence of GeO_2_ [73,74]. The residual stress of the core was found to be under a tension of 7.7 MPa, with the standard deviation of 1.5 MPa except the center of the core 17.5 MPa, but that of the cladding was a compression of −26 MPa. The stress in the core is normally positive (tensile) due to the external pulling force during the drawing process. However, the compressive stress in the cladding is developed for the balance of the forces after removing the drawing tension [75,76]. On the other hand, after the γ-ray irradiation at the total dose of 3570 Gy, no influence on the refractive index was observed. However, a change in the residual stress of the center of the core and the cladding was found. The tensile stress around the center of the core was relaxed from 17.5 MPa to 7.0 MPa with the slight change in tensile stress of 7.8 MPa in the outer core region (the standard deviation of 1.3 MPa). Further, the compressive stress in the cladding slightly increased from −26 MPa to −27 MPa.

**Figure 5:**
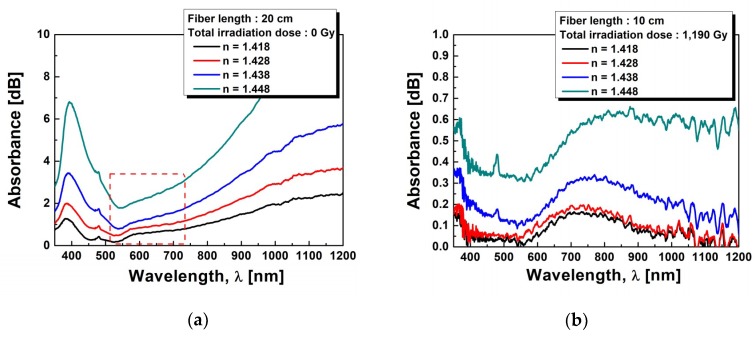

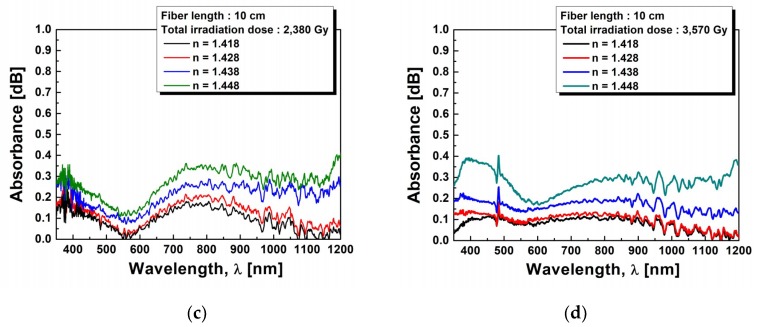
The SPR spectra obtained by dropping the matching oils of different refractive indices (**a**) before and after the γ-ray irradiation at the total dose of (**b**) 1190 Gy, (**c**) 2380 Gy, and (**d**) 3570 Gy.

To investigate the effect of the γ-ray irradiation on the SPR sensitivity, the SPR absorption of the Au NPs (cladding)-doped fiber was measured by dropping the index matching oils onto the stripped portions of 3 cm. The total fiber length was 10 cm and the refractive index (n = 1.418–1.448) of the matching oils was varied. Figure 5 shows the SPR spectra of the fiber before and after the irradiations of 1190 Gy, 2380 Gy, and 3570 Gy. Before the γ-ray irradiation, two SPR bands were found to appear, the first band around 380 nm and the second band around 580 nm [77]. The first SPR band shifted towards longer wavelength from 381.95 nm to 394.15 nm with the increase of the refractive index from 1.418 to 1.448. The intensity of the SPR band also increased. The baseline corrected absorption intensity of the SPR spectrum from 0.80 dB to 4.34 dB increased with the increase of the refractive index due to the difference in diffraction orders [62,78]. The full width at half maximum (FWHM) of the SPR band was broadened from 49.06 nm to 71.29 nm with the increase of the refractive index due to the spatial spreading and scattering of the conduction electrons [25,52,62,79]. The average SPR intensity and the average FWHM were 2.09 dB and 60.55 nm, respectively. However, the second SPR band wavelength ~580 nm, shown as the red dashed square in Figure 5a, was hard to define, and its appearance and the shift of the SPR band with the increase of the refractive index may be due to the low plasmon coupling of the aggregated Au NPs.

After the γ-ray irradiation, interestingly, another SPR band was found to appear around 750 nm. The intensity of this band increased with the increase of the index. However, after the γ-ray irradiation of 1190 Gy, the SPR band wavelength at 380 nm shifted towards shorter wavelength of 360 nm. Furthermore, at the higher radiation doses, it was hard to analyze the SPR bands at short wavelengths because the SPR band intensity decreased with the increase of the optical loss and noise. The SPR band wavelength at 360 nm slightly shifted towards longer wavelength with the increase of the refractive index. The amount of red shift of the SPR band around 750 nm with the increase of the refractive index was much larger than that of the SPR band at 360 nm and it was due to the interparticle plasmon coupling of the aggregated Au NPs compared to the single Au NPs of the 360 nm band. A more detailed description is given in the discussion section.

## 4. Discussion

From the results of TEM images and size distributions of Au NPs in the cladding of the fiber before the γ-ray irradiation as shown in Figure 2a, the surface density and volume density of the Au NPs in the cladding of the fiber were estimated to be about 23 × 10^3^ NPs/μm^2^ and 2,406 × 10^3^ NPs/μm^3^, respectively, with the aspect ratio of 1.00. As the total dose of the irradiation increased to 1190 Gy, 2380 Gy, and 3570 Gy, the average aspect ratio of the Au NPs increased to 1.05, 1.29, and 2.00, respectively. The size distribution of the short axis of the Au NPs also slightly increased with the increase of the γ-ray irradiation level. It is clear that the γ-ray irradiation induced the growth of Au NPs by the direct coalescence and consequent transformation from spherical to prolate in shape. The similar growth of crystalline Au NPs in silica glass due to Ostwald ripening by the ion irradiation was also reported [53].

Due to the morphological change of Au NPs in the fiber cladding after γ-ray irradiation, the intensity and the peak wavelength of the two SPR bands were found to change as shown in Figure 3. Before the irradiation, the separated SPR bands at 392 nm and 509 nm are due to the split of the plasmon resonance into a longitudinal SPR mode and a transverse SPR mode, parallel and perpendicular to the long axis of the Au NPs, respectively [80,81]. The short wavelength band at 392 nm is attributed to the transverse mode of SPR, which is assigned to the dipole resonance of the Au NPs. And it is much stronger than that at 509 nm, which is attributed to the longitudinal mode of SPR, due to the spherical Au NPs. The weaker absorption at 509 nm from the longitudinal mode of SPR may have some contribution from the interparticle plasmon coupling of the aggregated Au NPs. After the γ-ray irradiation, the clear appearance of the SPR bands indicates the significant morphological change of the Au NPs as shown in Figure 2. With the increase of the total dose of γ-ray irradiation, the longitudinal resonance at longer wavelength of 509 nm seems to be dominant. It shifted from 509 nm to 553 nm, 584 nm, and 597 nm with the increase of the total dose to 1190 Gy, 2380 Gy, and 3570 Gy, respectively. In addition, the absorption intensity increase with the increase of the total dose of γ-ray irradiation is thought to be due to the increase of the aggregated Au NPs. 

The unclear peak position of the first SPR band at 392 nm after the γ-ray irradiation may be due to the wavelength overlap from the absorptions of the radiation-induced defects. The absorption increase may be due to the radiation-induced defects. The possible defects are oxygen deficient centers (Si-ODC, ≡Si-Si≡ at 394 nm), per-oxy radicals (Si-POR, ≡Si-O-O at 630 nm), self-trapped hole defects (Si-STHs at 477 nm, 574 nm, 663 nm, 765 nm), non-bridging oxygen hole centers (Si-NBOHC, ≡Si-O·at 620 nm) due to strained Si-O bonds, GeX (at 475 nm), and Ge-NBOHC (≡Ge-O at 630 nm) due to strained Ge-O bonds [73,82,83,84,85,86,87]. 

Furthermore, the splitting of the SPR bands became more distinct with the increase of γ-ray irradiation dose. It is known that the anisotropically shaped metal particles show a split SPR band due to the transverse and longitudinal modes of charge density oscillations in the presence of electromagnetic radiation [10,11,53]. Thus, the splitting of the SPR bands may be due to the morphological change, especially the increase of the aspect ratio, of the Au NPs. As the total dose of γ-ray irradiation increased, the longitudinal SPR band at longer wavelength of 509 nm showed a tendency to shift towards longer wavelength due to the growth of Au NPs from spherical to prolate in shape as shown in Figure 3b [12,81].

In the case of the refractive index and the residual stress of the fiber, after the γ-ray irradiation at the total dose of 3570 Gy, the negligible change in the refractive index and the residual stress of the cladding by the γ-ray irradiation of the total dose of 3570 Gy does not contribute to the SPR sensing property.

From the results of the SPR sensing test by dropping the index matching oils onto the stripped portions of 3 cm as shown in Figure 5, the measured two SPR bands are related to the spherical Au NPs (transverse SPR mode) and the aggregated Au NPs (longitudinal SPR mode), respectively as known from the results shown in Figure 2 and Figure 3. Before the γ-ray irradiation, the SPR sensitivity (wavelength/refractive index unit, nm/RIU) of the first SPR band around 380 nm was estimated to be 406.7 nm/RIU. It is well known that the SPR band wavelength increase with the increase of the refractive index is related to the resonance wavelength of the incident light due to the increase of the wave vector of the surface plasmon mode [52,60,61,62,64,70]. After the γ-ray irradiation, the new SPR band around 750 nm may be due to the interparticle plasmon coupling of the aggregated Au NP, in good agreement with the TEM results as shown in Figure 2. The Au NPs was grown in size and morphologically changed to aggregates due to the bridge-like connection and crosslinking of the Au NPs by the γ-ray irradiation. However, the SPR band wavelength due to the spherical Au NPs blue-shifted form 380 nm to 360 nm because the shifts of the transverse and longitudinal SPR band are contra-directed [12,81]. 

**Figure 6:**
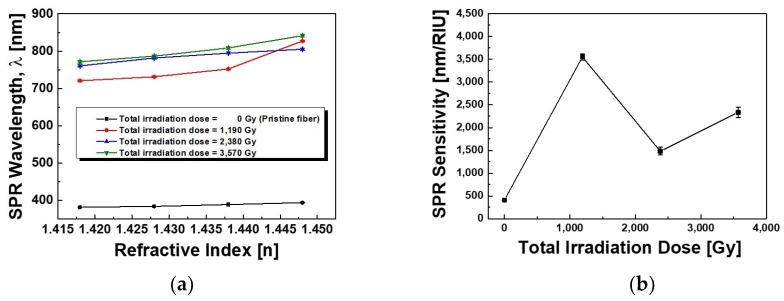

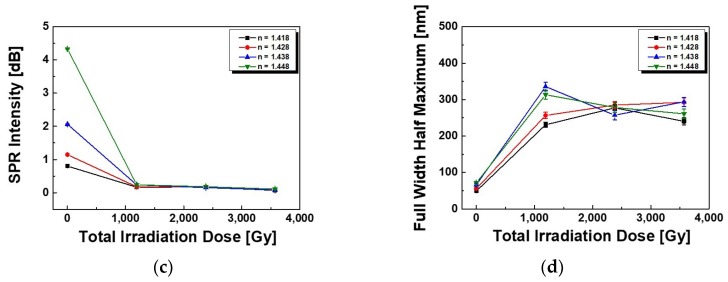
Variation of (**a**) the SPR wavelength, (**b**) the SPR sensitivity, (**c**) the SPR intensity, and (**d**) the FWHM of the SPR spectrum as a function of refractive index of the matching oils before and after the γ-ray irradiation at the total dose of 1190 Gy, 2380 Gy, and 3570 Gy.

Finally, the results of the SPR spectra shown in Figure 5 were replotted in Figure 6 for a better look and summary. The variation of the SPR band wavelength, the SPR sensitivity, the SPR intensity, and the FWHM of the SPR spectrum were shown as a function of refractive index of the matching oils under the γ-ray irradiation. The SPR band wavelength showed a tendency to shift towards longer wavelength with the increase of the total dose of γ-ray irradiation regardless of the corresponding refractive indices due to the growth and assembly of Au NPs into the Au NP clusters by the γ-ray irradiation (Figure 6a). From the results of the SPR spectra as shown in Figure 6a, the center wavelengths of the SPR band were found at 720.67 nm, 731.33 nm, 752.29 nm, and 827.27 nm with the increase of the refractive index 1.418, 1.428, 1.438, and 1.448 under at dose of 1190 Gy, respectively. And under the total dose of 2380 Gy and 3570 Gy, the SPR band wavelengths were found to appear at 760.75 nm, 781.71 nm, 794.77 nm, and 805.25 nm and at 771.53 nm, 786.70 nm, 808.65 nm, and 841.57 nm with the increase of the refractive index, respectively. The estimated sensitivities (wavelength/RIU) of the SPR sensor based on the γ-ray irradiated fibers on the sensing capability of refractive index (n = 1.418–1.448) increased to be 407 nm/RIU, 3553 nm/RIU, 1483 nm/RIU, and 2335 nm/RIU before and after the γ-ray irradiation at the total dose of 1190 Gy, 2380 Gy, and 3570 Gy, respectively, as shown in Figure 6b. 

After the γ-ray irradiation, the SPR sensitivity increased rapidly but saturated with the increase of the total dose of γ-ray irradiation. The baseline corrected SPR band intensity slightly increased from 0.17 dB to 0.24 dB, from 0.17 dB to 0.18 dB, and from 0.07 dB to 0.11 dB with the increase of the refractive index from 1.418 to 1.448 at the total dose of 1190 Gy, 2380 Gy, and 3570 Gy, respectively. The increase of the SPR intensity with the increase of the refractive indices, regardless of the γ-ray irradiation, is due to a leak of more divergent light beams from the cladding of the fiber [5,9,88]. However, as the total dose of γ-ray irradiation increased from 1190 Gy to 3570 Gy, the SPR band intensity decreased regardless of the corresponding refractive indices due to the increase of the radiation-induced loss as shown in Figure 6c. And the FWHM of the SPR band became broadened from 230.93 nm to 313.49 nm, from 277.47 nm to 278.52 nm, and from 240.48 nm to 260.96 nm with the increase of the refractive index from 1.418 to 1.448 at the total dose of 1190 Gy, 2380 Gy, and 3570 Gy, respectively. The broadened SPR band with the increase of the refractive indices regardless of the γ-ray irradiation is due to the spatial spreading and scattering of the conduction electrons and the FWHM of the SPR band was saturated with the increase of the total dose of γ-ray irradiation as shown in Figure 6d [25,62,79]. Note that the center wavelength, intensity, and the FWHM of the SPR band were rapidly changed after the γ-ray irradiation at the total dose of 1190 Gy as compared with the fiber before the γ-ray irradiation. However, the change in center wavelength, intensity, and the FWHM of the SPR band was saturated as the total dose of γ-ray irradiation of 2380 Gy and 3570 Gy increased regardless of the corresponding refractive indices. 

The SPR sensitivity, the average intensity, and the average FWHM of the fiber before and after the γ-ray irradiation at the total dose of 1190 Gy, 2380 Gy, and 3570 Gy are listed in Table 1. The average intensity and the FWHM were changed from 0.20 dB to 0.17 dB and 0.08 dB and from 284.21 nm to 274.59 nm and 272.03 nm at the total dose of 1190 Gy, 2380 Gy, and 3570 Gy, respectively. The average intensity and the FWHM of the SPR band around 750 nm in the range of the refractive index from 1.418 to 1.448 decreased with the increase of the total dose of γ-ray irradiation at 1190 Gy, 2380 Gy, and 3570 Gy.

From the SPR results of the fibers after the γ-ray irradiation, the growth of the Au NPs to anisotropically shaped Au clusters must have played an important role because it showed the splitting of the SPR band into the transverse and longitudinal modes [10,11,53]. The increase in the aspect ratio of the Au cluster led to red shifts of the longitudinal SPR band (longer wavelength) and it showed better sensitivity in response to changes in refractive index of the surrounding medium because the refractive index sensitivity of the SPR sensor was enhanced with increasing SPR band wavelength [89]. However, an excessive γ-ray irradiation is detrimental due to the decrease of the SPR sensitivity and the increase of the radiation-induced optical loss by excessively changing the size and shape of Au NPs. Nevertheless, the γ-ray irradiation of the SPR sensor fiber with an appropriate irradiation dose is an effective method to increase the SPR sensitivity and to control a desired detection wavelength for utilizing a commercially available light source and power detector.

## 5. Conclusions

The silica glass optical fiber incorporated with Au NPs in the germano-silicate glass cladding has been irradiated by ^60^Co γ-rays with the dose rate of 1190 Gy/h for 1 h to 3 h at room temperature to investigate the enhancement of the SPR sensitivity and the enabling tunability of the operation range of sensing wavelength of the SPR for the refractive index sensing by controlling the size and shape of the incorporated crystalline Au NPs in the fiber cladding. As the total dose of the γ-ray irradiation increased to 1190 Gy, 2380 Gy and 3570 Gy, the average aspect ratio of the Au NPs increased from 1.0 to 1.05, 1.29, and 2.00, respectively. The spherical Au NPs of ~3.8 nm diameter grew into the large clusters by the direct coalescence of NPs and then transformed to prolate shaped particles with a large aspect ratio. 

The anisotropically grown Au NPs with a large aspect ratio were the main cause of the splitting of the SPR bands into transverse and longitudinal modes, and this splitting became more distinct with the increase of γ-ray irradiation dose. Due to the effective shift and the intensity increase of the longitudinal SPR band at the longer wavelength of 509 nm after the γ-ray irradiation, the SPR sensitivity for the corresponding refractive indices (n = 1.418–1.448) increased rapidly but saturated from 406.7 nm/RIU to 3553 nm/RIU, 1483 nm/RIU, and 2335 nm/RIU with the increase of the total dose of γ-ray irradiation from 0 Gy to 1190 Gy, 2380 Gy, and 3570 Gy, respectively. However, the average SPR intensity decreased from 0.20 dB to 0.17 dB and 0.08 dB and the average FWHM of the SPR band around 750 nm also decreased from 284.21 nm to 274.59 nm and 272.03 nm with the increase of the total dose from 1190 Gy to 2380 Gy, and 3570 Gy, respectively. On the other hand, the transverse SPR band at 392 nm has not shown any significant increase in its intensity and wavelength shift. However, from the SPR sensing experiment using refractive index-matching oils after the γ-ray irradiation of 1190 Gy, the transverse SPR band at 380 nm was found to shift to 360 nm. 

No significant change in the refractive index and the residual stress after the γ-ray irradiation at the total dose of 3570 Gy indicates no contribution of them to the SPR sensing capability. 

## Figures and Tables

**Table 1:** The SPR sensitivity, the average intensity, and the average FWHM of the optical fiber embedded with Au NPs in cladding after the γ-ray irradiation.

γ-ray Irradiation	Total γ-ray Irradiation Dose [Gy]	Sensitivity [nm/RIU]	Average Intensity [dB]	Average FWHM [nm]
Before (SPR at 380 nm)	0	407	2.087	60.554
After (SPR at 750 nm)	1190	3553	0.204	284.207
	2380	1483	0.170	274.592
	3570	2335	0.084	272.026
